# Ni‐Co Bimetallic Catalysts Supported on Mixed Oxides (Sc‐Ce‐Zr) for Enhanced Methane Dry Reforming

**DOI:** 10.1002/open.202400086

**Published:** 2024-11-12

**Authors:** Ahmed E. Abasaeed, Ahmed A. Ibrahim, Anis H. Fakeeha, Mohammed O. Bayazed, Mabrook S. Amer, Jehad K. Abu‐Dahrieh, Ahmed S. Al‐Fatesh

**Affiliations:** ^1^ Chemical Engineering Department, College of Engineering King Saud University Riyadh 11421 Saudi Arabia; ^2^ Chemistry Department, College of Science King Saud University Riyadh 11451 Saudi Arabia; ^3^ School of Chemistry and Chemical Engineering Queen's University Belfast Belfast, Northern Ireland BT9 5AG UK

**Keywords:** Bimetallic Ni-CO catalyst, DRM, CO2-TPD, Mixed oxides (Sc, Ce, Zr) support, TGA

## Abstract

Dry methane reforming (DRM) presents a viable pathway for converting greenhouse gases into useful syngas. Nevertheless, the procedure requires robust and reasonably priced catalysts. This study explored using cost‐effective cobalt and nickel combined into a single catalyst with different metal ratios. The reaction was conducted in a fixed reactor at 700 °C. The findings indicate that the incorporation of cobalt significantly enhances catalyst performance by preventing metal sintering, improving metal dispersion, and promoting beneficial metal‐support interactions. The best‐performing catalyst (3.75Ni+1.25Co‐ScCeZr) achieved a good conversion rate of CH_4_ and CO_2_ at 46.8 %, and 60 % respectively after 330 minutes while maintaining good stability. The TGA and CO_2_‐TPD analysis results show that the addition of Co to Ni reduces carbon formation, and increases the amount of strong basic sites and isolated O_2_− species, and the total amount of CO_2_ desorbed. These results collectively highlight the potential of cobalt‐nickel catalysts for practical DRM applications and contribute to developing sustainable energy technologies.

## Graphical abstract

Ni−Co bimetallic catalysts supported on ScCeZr demonstrated promising performance for dry methane reforming (DRM) at 700 °C. The catalyst with a 3.75 : 1 Ni : Co ratio exhibited the highest CH_4_ conversion (46.5 %) due to its optimal balance of reducibility, CO_2_ interaction, and surface area. These findings highlight the importance of carefully tuning the metal composition to enhance DRM efficiency over ScCeZr‐based catalysts.

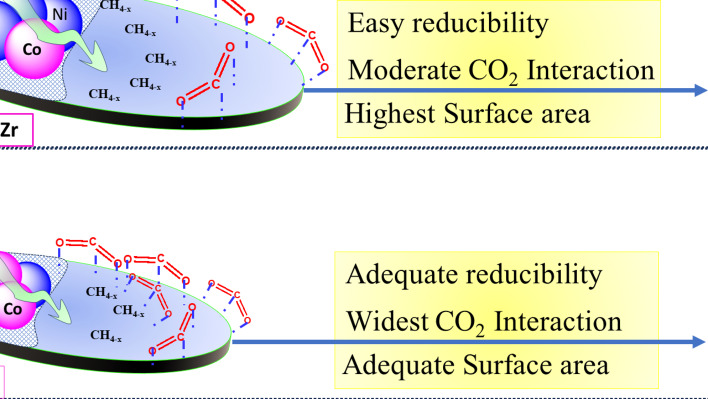

## Introduction

Novel approaches to combat climate change are necessary due to the increasing concentrations of methane and carbon dioxide in the atmosphere^.[1–3]^ This translates to dramatic shifts in seasonal patterns, relentless sea level rise, and ultimately, a devastating disruption to the delicate balance of ecological cycles and agricultural production.^[4]^ One method that shows promise for turning these greenhouse gases into useful syngas is the dry reformation of methane (DRM).^[5]^ This innovative process tackles the issue at its source, transforming the methane (CH_4_) and carbon dioxide (CO_2_) into valuable hydrogen‐rich syngas^.[6–8]^ 1

(1)

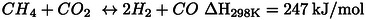




DRM provides a dual benefit: mitigating greenhouse gas emissions and producing clean hydrogen. However, the process’ high temperature requirements pose a challenge to catalyst stability. Oxide‐supported Ni and Co catalysts in the form of nanoparticles readily agglomerate (sinter), leading to a decrease in their active sites^.[9]^ The Equations (2)–(4) represent the side reactions of the process are water gas shift, methane cracking, and CO disproportionation respectively.

(2) $$H_{2}+{CO}_{2\;}↔H_{2}O+CO\;\Delta H298\;K=247\;\mathrm{kJ}/\mathrm{mol}$$

(3) $${CH}_{4}↔2H_{2}+C\;\Delta H298\;K=75\;\mathrm{kJ}/\mathrm{mol}$$

(4) $$2CO↔{CO}_{2}+C\;\Delta H298\;K=\text{-}172\;\mathrm{kJ}/\mathrm{mol}$$

Ni or Co‐based catalysts have been widely employed in the DRM reaction owing to their high activity, considerable availability, and low cost^.[10,11]^ In the DRM, the catalyst's activity and stability are highly dependent on the type of active metals employed. While Ni‐based catalysts boast high activity, they suffer from rapid deactivation at high temperatures due to a combination of sintering and excessive carbon deposition. In contrast, Co‐based catalysts not only maintain their activity and resist sintering at high temperatures but also offer a greater number of active sites on their surface compared to Ni catalysts^[12]^ While noble metals demonstrate superior activity and durability in DRM, their high cost precludes widespread industrial application.^[13]^ Bimetallic systems present a viable direction for future study despite the high cost and inherent drawbacks of precious metal and single‐metal catalysts.^[14]^ Therefore, combining cobalt (Co) and nickel (Ni) in a bimetallic catalyst represents an efficient strategy to exploit their synergistic effects and achieve reduced prices.^[15]^ While individual Ni and Co catalysts exhibit limitations in both activity and stability for the DRM reaction, incorporating a second metal into these monometallic catalysts offers a promising solution. This strategy aims to modify the electronic properties of cobalt and nickel sites to enhance catalytic activity and suppress carbon formation.^[16]^ Over the years, the combination of cobalt and nickel has been extensively used as active metals for DRM reactions. Cobalt provides redox ability to the Ni−Co bimetallic catalyst, thereby improving the catalyst's anti‐coking ability. The applications of bimetallic catalysts are proven to display a better catalytic performance than Ni or Co monometallic catalysts^[17]^ Research has identified optimal metal ratios for various catalyst supports. Furthermore, the bimetallic structure promotes alloy formation or synergistic interactions, which enhance metal dispersion on the support. Guo et al.; looked into the use of bimetallic nickel‐cobalt catalysts in the dry reforming of methane.^[18]^ They discovered that the support qualities show up in a variety of contexts, such as chemical and physical mutual effects. Redox, metal‐support, and acid‐base properties collectively influence catalyst chemical behavior. In the DRM reaction, Liang et al.; investigated the performance of the NiCo@C/Al_2_O_3_ catalyst. NiCo@C/Al_2_O_3_ prevented the development of coke and improved the oxidation of the carbon depositions. In comparison to individual Ni and Co catalysts.^[19]^ Luisetto et al.′s study showed that Ni−Co catalysts exhibit higher activity and coke resistance.^[20]^ CeO₂′s unique properties, including hydrogen spillover, enhance Co‐based catalyst performance and promote Ni−Co interaction, improving carbon resistance. Effective support selection is crucial for developing highly active Ni−Co catalysts for high‐temperature applications. Because metallic oxides are inexpensive and can disseminate and stabilize Ni−Co species—which is essential for high activity and decreased carbon production in bimetallic catalysts—they are the most researched supports.^[21]^ Rahemi et al.; studied the non‐thermal plasma‐assisted synthesis and physicochemical characterizations of Co and Cu‐doped Ni/Al_2_O_3_ nanocatalysts used for dry reforming of methane.^[22]^ They observed good interaction between the active metals and the support material. Especially, their activity measurements revealed that the Ni−Co/Al2O3 Nano catalysts exhibited superior performance compared to the undoped Ni/Al_2_O_3_ catalysts. Zirconia‐based supports are of interest due to their high‐temperature oxygen capacity,^[23]^ while CeO_2_ is distinguished for its oxygen storage and redox abilities in thermal and photocatalysis.^[24]^ On the other hand, Yun‐Chuan et al. studied the impact of Sc_2_O_3_ in Cu/ZrO catalysts on hydrogen production. They discovered that doping ZnO lattice with Sc^3+^ ions increased metal dispersion, and strengthened the interaction between Cu metal and the support oxide, leading to improved catalytic activity and stability of the Cu/Sc2O3‐ZnO catalyst.^[25]^ Combining different oxides can enhance the adsorption of active metal particles, improving catalytic activity.^[26]^ In this study, we addressed the limitations of single nickel or cobalt catalysts by enhancing their activity, durability, and performance in DRM. This was achieved by immobilizing Co−Ni nanoparticles on a support composed of mixed oxides (Sc, Ce, Zr). Optimal metal ratios for this support were determined for different catalyst formulations. X‐ray diffraction (XRD), surface area analysis, porosity study, temperature‐programmed reduction/oxidation/desorption (TPR, TPO, TPD), Raman spectroscopy, and thermogravimetric analysis (TGA) were among the methods used to thoroughly characterize the catalyst.

## Result and Discussions

### H_2_‐TPR and XRD

**Figure 1:**
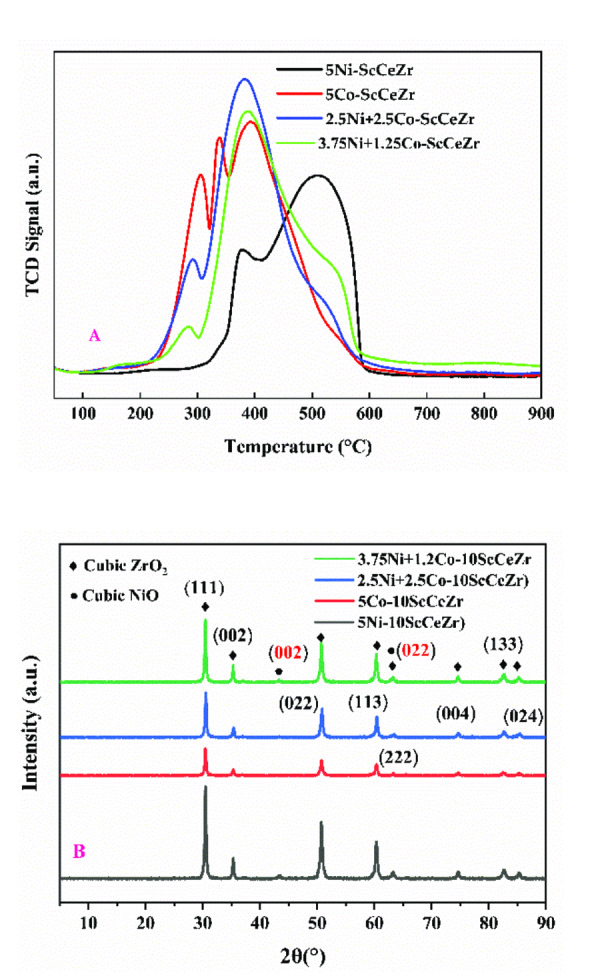
(A) H_2_‐Temperature Programmed Reduction profile of catalysts (B) The X‐ray diffraction pattern of Catalysts.

The H_2_‐temperature programmed reduction profile of Ni‐ScCeZr (5 % Ni), Co‐ScCeZr (5 % Co), 2.5Ni+2.5Co‐ScCeZr, and 3.75Ni+1.25Co‐ScCeZr catalysts is shown in Figure 1A. The various reduction peaks at different temperatures specify the extent of interaction of “NiO and CoO‐surface interacting species”. Reduction peaks observed below 300 °C suggest weak metal‐support interactions, while those between 300 and 400 °C indicate moderate interactions. As all reduction events occurred below 600 °C, subsequent reduction steps can be performed at or above this temperature.^[27]^ Peaks around 509 °C signify strong NiO‐support interactions.^[28]^ The Ni‐ScCeZr catalyst shows two interaction peaks: a moderate interaction at 377° and a strong interaction at 509°. The Co‐ScCeZr catalyst displays three moderate interaction peaks at 304°, 337°, and 393°. All are in the moderate strength zone. Bimetallic catalysts demonstrated enhanced reducibility with reduction peaks shifted towards lower temperatures. For example, the equimolar catalyst exhibited weak metal‐support interactions at 292 °C and moderate interactions at 383 °C. Similarly, the 3.75Ni+1.25Co‐ScCeZr bimetallic catalyst gives a small “the weakest strength” peak at 286° and a moderate strength peak at 387°. Bimetallic catalysts demonstrate enhanced reducibility, indicated by reduction peaks shifting to lower temperatures. TPR results indicate that adjusting Ni and Co content can facilitate reduction at lower temperatures, which is beneficial for DRM processes. Table 1 displays the hydrogen H₂‐consumption capacities and the corresponding percentage reductions for various catalysts. Markedly, the 3.75Ni+1.25Co‐ScCeZr bimetallic catalyst stands out as the most active among the group. This exceptional activity translates to the lowest H₂ consumption observed (17.570 cm^3^/g STP) and consequently, the lowest percentage reduction (92 %). Figure 1B presents the X‐ray diffraction (XRD) patterns of the catalysts: Ni‐ScCeZr (5 % Ni), Co‐ScCeZr (5 % Co), 2.5Ni+2.5Co‐ScCeZr, and 3.75Ni+1.25Co‐ScCeZr. These patterns offer valuable insights into the crystalline structure of the materials. Figure 1B provides valuable information about the crystalline nature of the studied catalysts. While NiO and ZrO_2_ phases are identified, the XRD patterns indicate that the other components might be present in a less crystalline form or that the abundant ZrO2 masks their signal. The patterns disclose the presence of individual metal oxide phases corresponding to cubic NiO (identified by peaks at 2θ=43.3° and 63.2°) and cubic ZrO_2_ (identified by multiple peaks at 2θ values of 30.5°, 35.1°, 50.6°, 54°, 60.3°, 63.2°, 74.3°, 82.2°, and 85.3°). These identifications are based on comparisons with standard reference patterns (JCPDS reference numbers 00–001–1239 for NiO and 00–027–0997 for ZrO_2_).

**Table 1:** H_2_‐consumptions in the TPR analysis and % reductions of catalysts

Samples	T (°C)	Quantity (cm^3^/g.STP)	Total H_2_ consumption (cm^3^/g STP)	Degree of Reduction^[a]^ (%)
5Ni‐ScCeZr	372 509	1.718 16.252	17.972	94
5Co‐ScCeZr	306 339 394	4.765 3.498 15.108	23.371	123
2.5Ni+2.5Co‐ScCeZr	288 382	0.657 23.695	24.352	128
3.75Ni+1.25Co‐ScCeZr	282 389	0.249 17.321	17.570	92

[a] Degree of Reduction^a^ (%)=(H_2_ consumption during H_2_‐TPR/theoretical H_2_ required to complete the reduction of active phases

### N_2_ Adsorption‐Desorption

**Figure 2:**
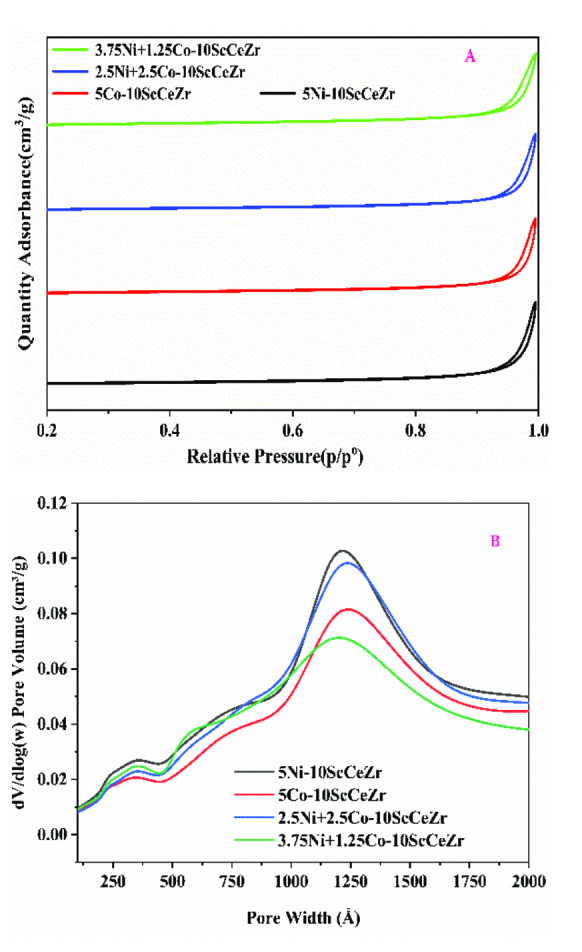
depicts (A) N_2_ adsorption‐desorption isotherms and (B) BJH distribution of catalyst samples.

The adsorption‐desorption plot and porosity distribution of Ni‐ScCeZr (5 % Ni), Co‐ScCeZr (5 % Co), 2.5Ni+2.5Co‐ScCeZr, and 3.75Ni+1.25Co‐ScCeZr catalysts are displayed in Figure 2A. Their respective data for surface area, pore volume, and pore diameter are presented in Table 2. Catalyst shows types IV adsorption isotherm with an H3 hysteresis loop, which is typical of a mesoporous structure with slit‐shaped pores of various sizes and shapes.^[29]^ The pores size distribution of catalysts determined by the Barrett‐Joyner‐Halenda formula (BJH) is monomodal in the 121.6 nm range as shown in Figure 2B.

**Table 2:** The catalysts’ texture characteristics.

Sample	BET Surface area (m^2^/g)	Pore‐volume (cm^3^/g)	Pore‐size (Å)
Ni‐ScCeZr	9.0	0.06	275.0
Co‐ScCeZr	8.6	0.05	260.3
2.5Ni+2.5Co‐ScCeZr	8.4	0.05	282.0
3.75Ni+1.25Co‐ScCeZr	9.2	0.05	274.4

All catalysts exhibited a consistent average pore size of approximately 28 nm, but surface areas were generally low. The top‐performing catalyst (3.75Ni+1.25Co‐ScCeZr) displayed the highest surface area of 9.2 m^2^/g. Pore volume remained relatively constant across all samples at around 0.05 cm^3^/g.

### CO_2_‐TPD

**Figure 3:**
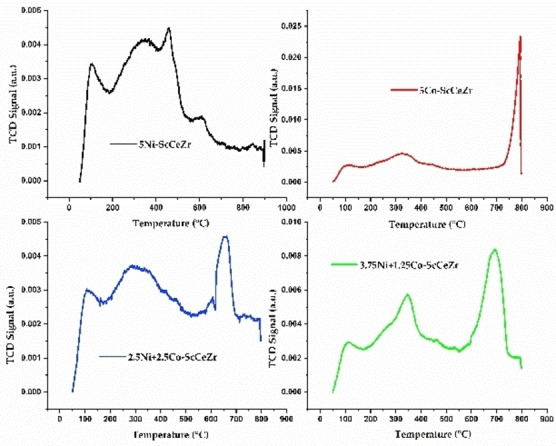
CO_2_‐TPD profiles of fresh catalyst samples.

Enhancing methane conversion in dry reforming requires a thorough understanding of the interactions between catalysts and CO_2_. Figure 3 exhibits the CO_2_‐TPD basic profiles of Ni‐ScCeZr (5 % Ni), Co‐ScCeZr (5 % Co), 2.5Ni+2.5Co‐ScCeZr, and 3.75Ni+1.25Co‐ScCeZr catalysts. CO₂‐TPD analysis indicated strong CO₂ adsorption at moderate temperatures for the Ni‐ScCeZr catalyst, suggesting favorable sites for methane activation. Conversely, the Co‐ScCeZr catalyst exhibited weaker CO₂ adsorption at higher temperatures. Bimetallic catalysts (2.5Ni+2.5Co‐ScCeZr and 3.75Ni+1.25Co‐ScCeZr) displayed a broader range of CO₂ adsorption sites, potentially enhancing interaction possibilities. It's important to note that the ability to adsorb increases with the Co content, as indicated in Table 1S. Of all the catalysts examined, the Ni‐ScCeZr and 3.75Ni+1.25Co‐ScCeZr catalysts showed the lowest total amount of CO_2_ desorbed during TPD analysis. This implies that they have the highest activity due to a well‐balanced combination of CO_2_ adsorption strength and distribution, resulting in efficient conversion of methane in dry reforming.

### Catalyst Activity

**Figure 4:**
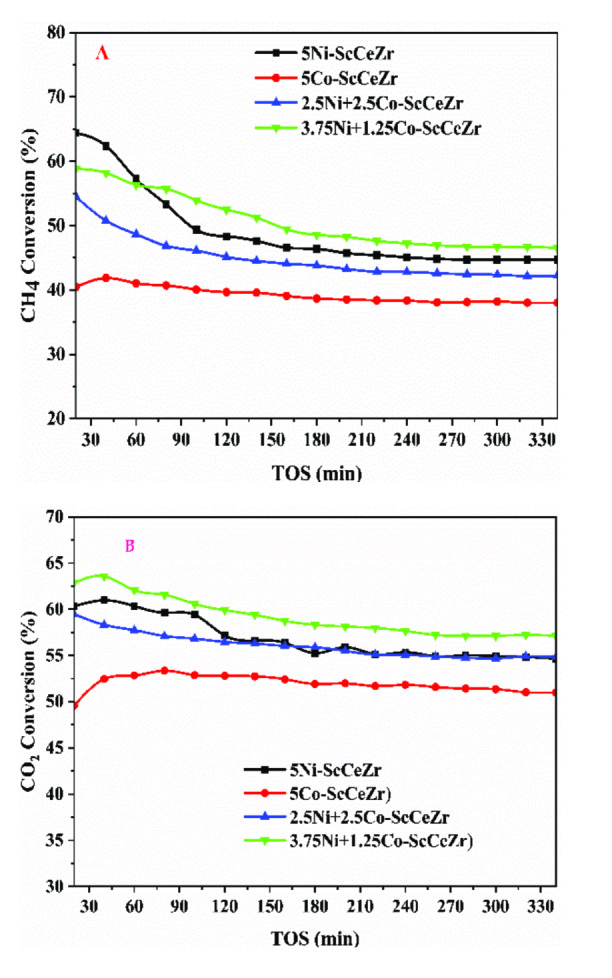
(A) Methane, (B) Carbon dioxide conversions of fresh catalysts at 700 °C, and 42000 mL(hg_cat_)^−1^.

Figure 4 displays the result of the catalytic DRM using both mon and bimetallic Ni−Co catalysts over 330 minutes. The reaction was conducted at atmospheric pressure and 700 °C while the feed gas of CH_4_ and CO_2_ was kept at a space velocity of 42,000 mL/(hg_cat_). The results illustrate the impact of Co loading on the bimetallic catalysts. CH_4_ conversion rates of 48.5 %, 45.9, 43.4, and 38.5 % were achieved for 3.75Ni+1.25Co‐ScCeZr, Ni‐ScCeZr, 2.5Ni+2.5Co‐ScCeZr, and Co‐ScCeZr respectively. Initial catalyst deactivation within the first two hours was attributed to carbon deposition on active sites. The 3.75 %Ni+1.25 %Co‐ScCeZr catalyst exhibited the highest overall activity, likely due to a combination of factors. Its performance was enhanced by superior reducibility (TPR), high surface area (textural analysis), and balanced CO₂ adsorption (CO₂‐TPD). It is worth noting that TGA analysis suggests that the Ni/Co ratio of 3.75/1.25 could be crucial in determining parameters such as CO_2_ adsorption and carbon production. This emphasizes how crucial it is to maintain a balanced Ni/Co ratio and optimize catalyst features to attain peak performance. In terms of CH_4_ conversion at various reaction and catalytic parameters, such as reaction temperature and time on stream (TOS), a comparison was done between the catalytic activity of the current work and that in the literature. Table 3 illustrates that results from the current catalysts were determined to be comparable with those reported in the literature.

**Table 3:** Compares the outcomes of this work with previously published results on supported bimetallic catalysts.

Catalysts	Trx (°C)	TOS (h)	Conversion (%)	Ref.
Ni−Fe/La_2_O_3_	700	50	43	[30]
10Ni+2 W/SBA‐15 A(IM)	700	2.8	32	[31]
NiCo/h‐BN	750	2	83	[32]
30 %Ni–30 %Fe/Al_2_O_3_	700	3.3	47	[33]
NiCo/MMAl	750	32	73–75	[34]
Ru+Co/CeO_2_	700	24	65	[35[
10Ni+10Co/SBA‐15	700	12	63	[36]
3.75 %Ni+1.25 %Co‐ ScCeZr	700	5.5	48.5	This work

Trx=Reaction Temperature.

### TGA Analysis

**Figure 5:**
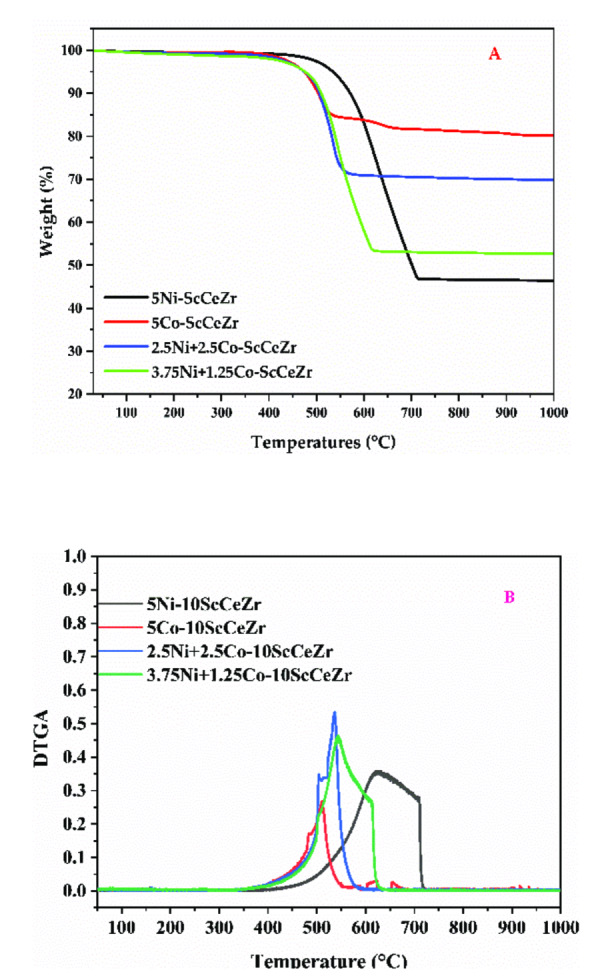
(A) The weight loss of the carbon deposits, (B) DTGA over spent catalysts.

The thermogravimetric measurement of the spent catalysts is shown in Figure 5A. The lowest level of amorphous carbon deposition is indicated by the minor weight loss below 450 °C.^[37]^ Conversely, the significant decrease between 500 °C and 750 °C suggests the presence and subsequent combustion of carbon nanotubes (CNTs).^[38]^ The Ni‐ScCeZr catalyst showed the most weight loss (54 %), according to TGA analysis. This is probably because of considerable carbon deposition, which is consistent with the catalyst's high activity and stability. On the other hand, the Co‐ScCeZr catalyst had the least amount of weight reduction (20 %), which aligns with its observed low activity. The intermediate weight losses of 30 % and 47 % for the 2.5Ni+2.5Co‐ScCeZr and 3.75Ni+1.25Co‐ScCeZr catalysts, respectively, show an interesting trend wherein increased Co loading in Ni−Co supported catalysts correlates with decreasing carbon production. The derivative thermogravimetric (DTG) curve in Figure 5B reveals that the greatest weight loss happens between 511 and 644 °C. This knowledge is essential for comprehending the connection between carbon production and catalyst composition, which permits catalytic performance to be improved.

### Raman Analysis

**Figure 6:**
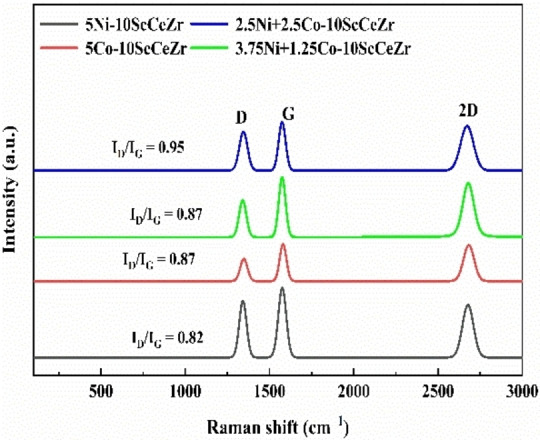
The spent Raman spectra of the supported samples.

Raman spectroscopy is a valuable technique for understanding the quality and crystallinity of carbon nanomaterials formed on the surface of the spent catalysts.^[39]^ Figure 6 depicts the typical Raman spectra of the spent catalysts, revealing three prominent peaks at 1345 cm^−1^, 1574 cm^−1,^ and 2671 cm^−1^, assigned to the D, G, and 2D bands, respectively. The D band signifies the presence of disordered carbon structures like defects and amorphous carbon,^[40]^ while the G band indicates highly organized, graphitic crystalline structures.^[41]^ The 2D Raman band originates from a second‐order scattering process involving two phonons. The I_D_/I_G_ ratio, indicative of material crystallinity, was lower in our samples, suggesting a higher degree of order. This is further supported by the presence of carbon species with a predominantly graphitic structure. Of all the catalysts, Ni‐ScCeZr demonstrated the lowest I_D_/I_G_ ratio, approximately 0.82, signifying a high degree of crystallinity and graphitization in the resulting carbon nanotubes. In contrast, the equimolar bimetallic catalyst displayed the highest I_D_/I_G_ ratios (0.95), suggesting decreased crystallinity due to a lack of graphitized carbon content. This indicates the incorporation of Co into Ni−Co/ScCeZr catalysts resulted in lowered degree of graphitization during the carbon formation process.

### TEM Analysis

**Figure 7:**
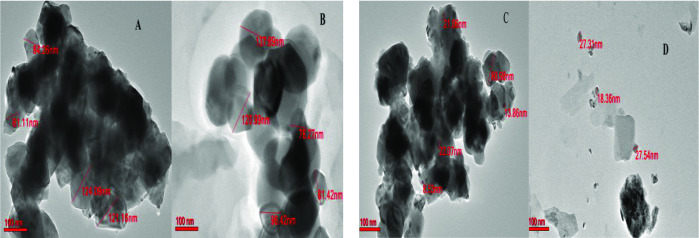
displays the TEM images of Ni‐ScCeZr catalyst for fresh (A), and used (B) and 3.75Ni+1.25Co‐ScCeZr samples for fresh (C), and used (D).

Transmission Electron Microscopy analysis was used to study the structure and composition of the catalysts. Figures 7(A) and (B) show the corresponding images for the mono catalyst Ni‐ScCeZr. The nickel particles are visible throughout the material in both the fresh and used samples. The particle size in the fresh Ni‐ScCeZr catalyst ranged from 61.1 to 124 nm, and it slightly increased to a range of 78.2 to 137 nm after being used. Similarly, Figure 7(C) shows the fresh 3.75Ni+1.25Co‐ScCeZr catalyst, while Figure 7(D) shows the same catalyst after being used. Both fresh and used samples exhibited relatively uniform Ni and Co particle distributions, though the used sample demonstrated a more consistent dispersion. Particle size analysis indicated a broader range (8.5–80.9 nm) for the fresh catalyst compared to the used catalyst (18.3–27.0 nm).

**Figure 8:**
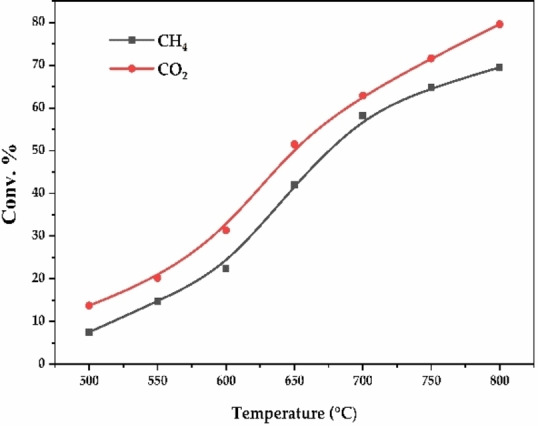
The effect of reaction temperature on conversion for the 3.75Ni+1.25Co‐ScCeZr.

Figure 8 investigates how temperature affects the conversion of methane (CH_4_) and carbon dioxide (CO_2_) for the optimal bimetallic catalyst (3.75Ni+1.25Co‐ScCeZr). As the reaction temperature increases from 500 to 800 °C, conversion rates for both CH₄ and CO₂ rise. This enhancement is attributed to the endothermic nature of the DRM process. Elevated temperatures provide reactant molecules with greater kinetic energy, facilitating collisions and promoting bond breaking, thus increasing product formation. The Arrhenius equation (Equation (5)) allows us to calculate the activation energy (E_a_) for methane and carbon dioxide conversion using reaction temperature data and corresponding conversion rates. This equation relates the rate constant (k), pre‐exponential factor (A), activation energy (Ea), universal gas constant (R), and temperature (T).
(5)

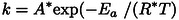




We obtain a linear relationship by rearranging the Arrhenius equation, taking the natural logarithm (Equation (6)), and plotting ln(k) versus 1/T yields a slope of −E_a_/R. From this slope, the activation energy (E_a_) can be calculated using E_a_=−slope * R.

(6) $$Ln\left(k\right)=\ln A+{(-E_{a}/}_{R})/\left(T\right)$$

**Figure 9:**
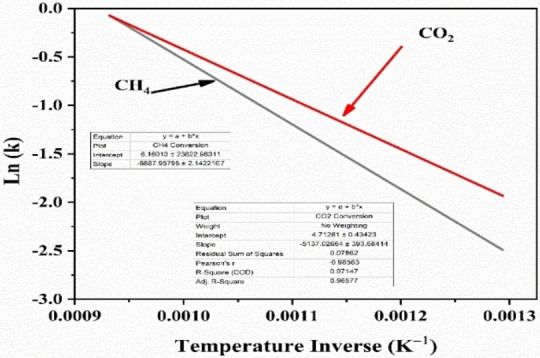
Arrhenius plot for the calculation of the activation energies (E_a_) of CH_4_ and CO_2._

Table 4 summarizes the rate constants derived from the given conversions at different temperatures. These values can be used in the aforementioned method to determine the activation energy for both CH_4_ and CO_2_ conversion. Activation energies for CH_4_ and CO_2_ conversion were estimated from the Arrhenius plot in Figure 9. Approximate values of 55,603 J/mol and 42,709 J/mol, respectively, were determined based on the data presented in Figure 8.

**Table 4:** Presents the rate constants obtained from the experiments at various temperatures.

T (°C)	T(K)	1/T(K)	Conv. CH_4_	Ln(k)	Conv. CO_2_	Ln(k)
500	773	0.0013	0.075	−2.5903	0.137	−1.9878
550	823	0.0012	0.147	−1.9173	0.202	−1.5995
600	873	0.0011	0.2242	−1.4952	0.3135	−1.1599
650	923	0.0011	0.4209	−0.8654	0.515	−0.6636
700	973	0.0010	0.582	−0.5413	0.629	−0.4636
750	1023	0.0010	0.6473	−0.4349	0.716	−0.3341
800	1073	0.0009	0.695	−0.3638	0.7963	−0.2278

## Experimental

### Materials and Methods

The following materials were used in the preparation of the desired catalysts; nickel nitrate hexahydrate (Ni (NO_3_)_2_.6H_2_O, ≥99 % Sigma Aldrich), cobalt nitrate hexahydrate (Co (NO_3_)_2_.6H_2_O; ≥99.0 %, Sigma Aldrich), and ScCeZr support consisting of 11 wt. % Sc_2_O_3_, 1.5 wt.% Ce, and 87.5 wt.% ZrO_2_ obtained from Japan

### Catalyst Preparation

#### Preparation of Bimetallic Ni and Co Supported on ScCeZr

Four catalysts were synthesized: Ni‐ScCeZr (containing 5 % Ni), Co‐ScCeZr (containing 5 % Co), and two bimetallic variations, 2.5Ni+2.5Co‐ScCeZr and 3.75Ni+1.25Co‐ScCeZr. Their metal precursors (hydrated Ni/Co nitrate) were dissolved in water, impregnated onto ScCeZr support at 80 °C, dried overnight at 120 °C, and finally calcined at 600 °C for 3 hours, achieving supported single metal and bimetallic catalysts with varying metal ratios. The catalyst's characterization (S1) and activity performance (S2) have been included in the supplementary information file.

## Conclusions

This study established the potential of Ni−Co bimetallic catalysts supported on ScCeZr for efficient dry methane reforming (DRM. These catalysts were prepared using wet impregnation and tested at 700 °C in a fixed‐bed reactor. By optimizing the Ni and Co ratio, a catalyst with high activity and stability was achieved. The significance of low‐temperature reducibility for optimal performance was highlighted by TPR analysis. Furthermore, the research found that ideal CO_2_ adsorption strength and distribution are crucial for effective catalyst performance, as demonstrated by the findings in the CO_2_‐TPD study. The catalyst containing 1.25 % Co and 3.75 % Ni performed better than the other catalysts. Characterization studies, however, showed a trend toward carbon deposition, especially as the Ni content increased. This indicates a possible deactivation mechanism but also emphasizes how crucial it is to balance catalytic activity and carbon resistance. Analysis of the deposited carbon revealed highly graphitized and crystalline structures, and Raman spectra indicated a relationship between the concentration of Ni and carbon production observed during TGA analysis. The relationship between CO₂ adsorption strength and catalytic activity was further demonstrated by the CO₂‐TPD data, where catalysts with moderate CO₂ adsorption performed better. The study highlights the importance of optimizing the composition and characteristics of these catalysts to achieve better performance in methane dry reforming.

## Conflict of Interests

There is no conflicting interest

1

## Supporting information

As a service to our authors and readers, this journal provides supporting information supplied by the authors. Such materials are peer reviewed and may be re‐organized for online delivery, but are not copy‐edited or typeset. Technical support issues arising from supporting information (other than missing files) should be addressed to the authors.

Supporting Information

## Data Availability

The data that support the findings of this study are available from the corresponding author upon reasonable request.
