# Supplementary material for: Ni‐Co Bimetallic Catalysts Supported on Mixed Oxides (Sc‐Ce‐Zr) for Enhanced Methane Dry Reforming
Source: ChemistryOpen. 2024 Nov 12;13(12):e202400086. doi: 10.1002/open.202400086 (PMC11625958; doi:10.1002/open.202400086)
Supplement: Supplementary file 1 — Supporting Information [file OPEN-13-e202400086-s001.pdf]

ChemistryOpen

Supporting Information

Ni-Co Bimetallic Catalysts Supported on Mixed Oxides (Sc-Ce-Zr) for Enhanced Methane Dry Reforming

Ahmed E. Abasaeed, Ahmed A. Ibrahim, Anis H. Fakeeha, Mohammed O. Bayazed, Mabrook S. Amer, Jehad K. Abu-Dahrieh,* and Ahmed S. Al-Fatesh*

Catalyst characterization S1

X-ray diffraction (XRD) investigation of catalyst samples was performed by Rigaku diffractometer using Cu Kα radiation source operated at 40 kV and 40 mA. 0.01step size and 5–100 scanning range was set for analysis. Phase analysis were performed by using X'pert high score plus software matched with JCPDS database. N_2-physiosorption isotherms investigation of catalyst sample was carried over Micromeritics Tristar II 3020. Surface area was assessed by Brunauer-Emmet Teller (BET) method whereas pore volume and pore diameter were assessed by Barrett-Joyner-Halenda (BJH) method. Reducibility of catalyst sample was investigated by H2-temperature-programmed reduction (TPR) over Micromeritics Auto Chem II 2920, USA. 70 mg of the sample was subjected to a heat treatment at 10 °C/min up to 900 °C under 30 mL/min gas flow of 10% H_2/Ar mixture gas. The thermogravimetric analysis (TGA) was done over 0.015 g of spent catalyst sample in the temperature range (room temperature to 1000°C) at heating ramp 20 °C by using Shimadzu TGA-51. The TGA analysis was carried out under oxidizing gas O_2. The weight loss/gain of catalyst sample against temperature was monitored continuously. O_2-Temperature programmed oxidation (TPO) was done on spent catalyst system over a temperature range of 50–800 °C by using 10% O_2/He mixture through by Micromeritics AutoChem II. Before analysis, the spent catalyst was treated under high purity Argon at 150 °C for 30 min and subsequently cooled to room temperature. The morphology of the catalyst sample was investigated by using a field emission scanning electron microscope (FE-SEM, model: JEOL JSM-7100 F) and transmission electron microscope (TEM, model: 120 kV JEOL JEM-2100F).

Catalyst activity test S2

The detail reaction set up for dry reforming reaction is shown in Figure S1. Catalytic dry reforming of methane was carried out over 0.1 g catalyst packed in fixed-bed stainless steel tubular micro-reactor (PID Eng & Tech micro activity reference company; L= 30cm, I.D = 9.1 mm) at atmospheric pressure. An axially positioned K type stainless steel sheathed thermocouple at the centre of the catalyst bed monitored the temperature within the reactor. Prior to the reaction, catalyst was activated under 40ml/min flow of H2 for 60 min at 700oC followed by purging of N2 for 15 min to remove the remnant of H2. Further, temperature of reactor was raised to 700°C under flow of N2. 30 ml/min CH4 and 30 ml/min of CO2 and 10 ml/min N2 (total flow rate of feed gas 70 ml/ min) was allowed to pass through the catalyst bed at 700 °C at equivalent space velocity of 42000 ml/hgcat. GC-2014 SHIMADZU (Column: Shin carbon C20380 for gases and Haysepe Q AC0209 column for water analysis; carrier gas: Argon) equipped with conductivity detector was used to analyse the feed and output gas composition. The expression for CH4 and CO2 conversions and is given as:

$$\% \, CH_4 \text{ conversion} = \frac{CH_{4,in} - CH_{4,out}}{CH_{4,in}} \times 100 \quad (S5)$$

$$\% \, CO_2 \text{ conversion} = \frac{CO_{2,in} - CO_{2,out}}{CO_{2,in}} \times 100 \quad (S6)$$

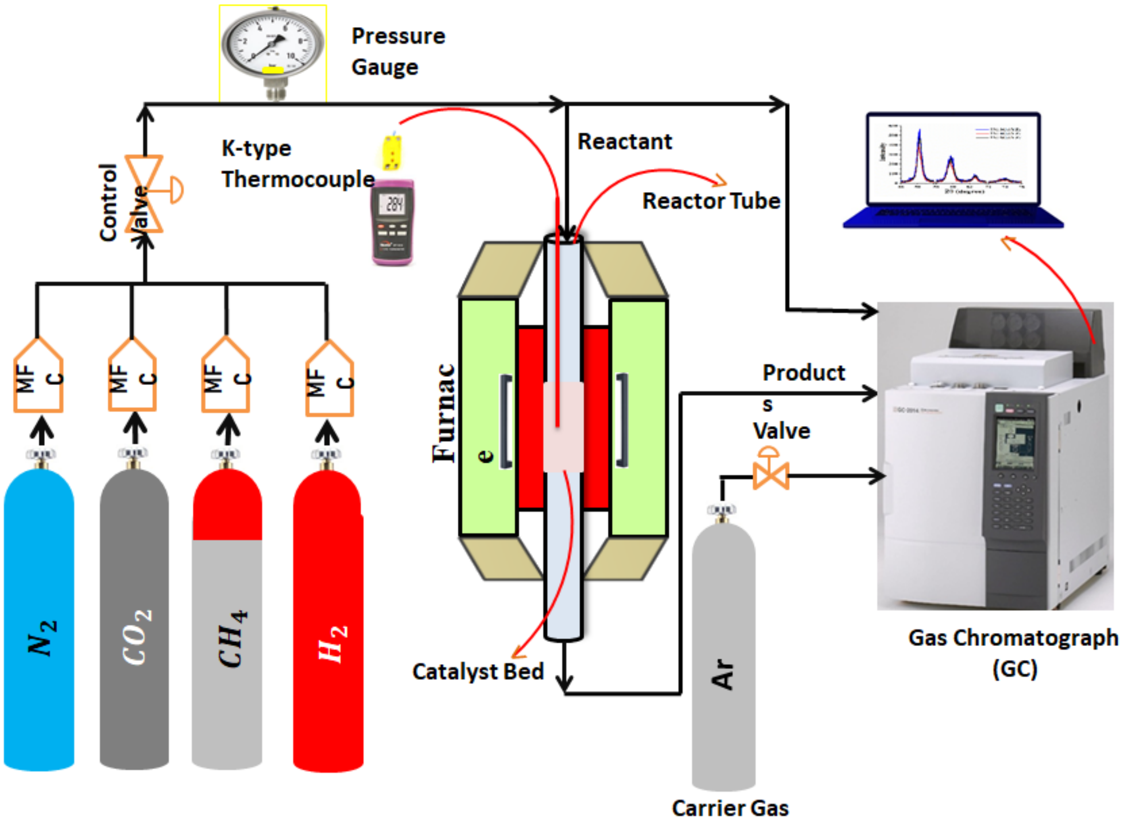

Figure S1. Reaction set up for dry reforming reaction

Table 1S CO_2 desorption during the TPD

Samples	Temperature at maximum (°C)	Quantity CO_2 Desorbed (cm^3/gSTP)	Total Quantity CO_2 Desorbed (cm^3/gSTP)
Co-ScCeZr	93	0.079	2.007
	326	0.141	
	795	1.787	
Ni-ScCeZr			0.44
	99	0.154	
	310	0.140	
	460	0.073	
	610	0.026	
	894	0.047	
2.5Ni+2.5Co-ScCeZr	103	0.099	1.362
	348	0.374	
	695	0.889	
3.75Ni+1.25Co-ScCeZr			0.503
	106	0.074	
	283	0.190	
	421	0.004	
	464	0.008	
	607	0.015	
	662	0.198	
	794	0.014	
